# How Clinical and Radiological Findings in Chronic Mandibular Osteomyelitis Do Not Always Correlate: Diagnostic Dilemmas in Dental-Related Bone Inflammations

**DOI:** 10.3390/diagnostics16101427

**Published:** 2026-05-07

**Authors:** Kamil Nelke, Ömer Uranbey, Ece Gülbağ, Büşra Ekinci, Burcu Gürsoytrak, Angela Rosa Caso, Michał Gontarz, Maciej Janeczek, Piotr Kuropka, Maciej Dobrzyński

**Affiliations:** 1 Maxillo-Facial Surgery Ward, EMC Hospital, Pilczycka 144, 54-144 Wrocław, Poland; 2 Academy of Applied Sciences, Health Department, Academy of Silesius in Wałbrzych, Zamkowa 4, 58-300 Wałbrzych, Poland; 3 Department of Oral and Maxillofacial Surgery, Faculty of Dentistry, Aydın Adnan Menderes University, Aydın 09100, Türkiye; 4 Department of Medical Pathology, Faculty of Medicine, Aydın Adnan Menderes University, Aydın 09100, Türkiye; 5 Department of Oral and Maxillo-Facial Surgery, University of Siena, Viale Aldo Moro, 2-Siena SI, 53100 Siena, Italy; 6 Department of Cranio-Maxillo-Facial Surgery, Maxillo-Facial Surgery Clinic, University Hospital in Cracow, Macieja Jakubowskiego 2 Street (Nowy Prokocim), 30-688 Krakow, Poland; 7 Department of Biostructure and Animal Physiology, Wrocław University of Environmental and Life Sciences, Kożuchowska 1, 51-631 Wrocław, Poland; 8 Division of Histology and Embryology, Department of Biostructure and Animal Physiology, Wrocław University of Environmental and Life Sciences, Cypriana K. Norwida 25, 50-375 Wrocław, Poland; piotr.kuropka@upwr.edu.pl; 9 Department of Pediatric Dentistry and Preclinical Dentistry, Wrocław Medical University, Krakowska 26, 50-425 Wrocław, Poland

**Keywords:** osteomyelitis, mandible bone, inflammation, chronic bone inflammation, Garre osteomyelitis, fibro-osseous lesions

## Abstract

The range of possible inflammatory changes in the oral cavity and in the maxillary and mandibular bones may present with diverse patterns and characteristics in both clinical and radiological evaluation. In most cases, a standard radiological examination, such as dental panoramic radiograph (DPR), has significant limitations in assessing early or complex bone changes associated with chronic bone inflammation. Advanced imaging with multidetector computed tomography or cone-beam computed tomography (MDCT or CBCT) can improve lesion characterization and surgical planning when a detailed evaluation of tooth-bearing structures, tooth apices, cortical plates, and cancellous bone is required. Such imaging allows more detailed assessment of alterations in medullary bone morphology and architecture, as well as identification of possible periosteal reactions adjacent to chronic bone inflammation. Osteomyelitis of the jaws comprises a heterogeneous group of inflammatory bone disorders characterized by variable clinical presentations and a broad spectrum of radiological appearances. Depending on disease chronicity, host factors, and microbial burden, mandibular osteomyelitis may mimic odontogenic tumors, fibro-osseous lesions, or malignant bone pathologies. Quite often, dental treatment affects bone status and condition, leading to unwanted events such as bone inflammation. Imaging plays a central role in diagnosis; however, radiographic findings are often nonspecific, particularly in early or chronic stages. Each case of osteomyelitis underscores the importance of correlating imaging findings with clinical history and highlights the role of repeated imaging in distinguishing inflammatory bone disease from aggressive jaw lesions. This study aims to characterize diverse patterns of chronic mandibular osteomyelitis associated with various prior treatment modalities using CBCT. By presenting a series of illustrative cases from heterogeneous clinical settings, the authors highlight the nonspecific radiographic features and diagnostic challenges inherent in chronic bone inflammation. The focus remains on the interpretation of complex imaging findings rather than a comparative analysis of technical protocols.

**Figure 1:**
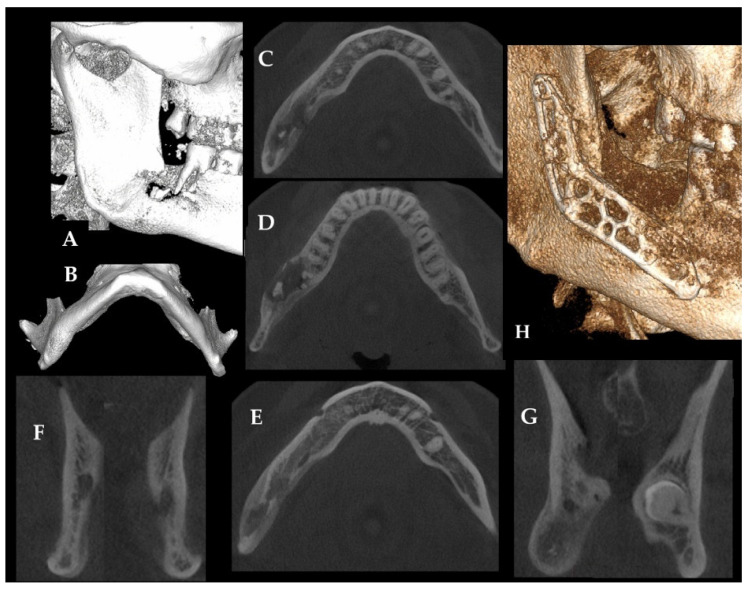
Delayed mandibular fracture and chronic osteomyelitis after impacted third molar extraction. The occurrence of local bone inflammation and osteomyelitis (OM) and even its chronic form might be caused by a variety of factors. Any dental procedures, iatrogenic complications, unwanted odontogenic infections, cases related to trauma, and the occurrence of early and late pathological or iatrogenic fractures and even other factors might lead to a chronic osteomyelitis. Both teeth/dental, sinus, skin-related, or other sources of inflammation might affect the bone status, condition, and occurrence of OM within the jaw bones [1,2,3,4,5]. The variety of cases are not identified in time and are treated insufficiently, leading to bone OM spread and a decrease in patients’ local status. Quite often, insufficient dental treatment like endodontic approaches or teeth removal without special care for the adjacent bone, soft tissues, and the alveolar area effects the possible occurrence of other dental-related, odontogenic infections, which might cause great clinical and radiological challenges in evaluating the most accurate approach to each case. When an impacted tooth is removed, the occurrence of immediate mandibular fracture is a rare complication. On the other hand, a pathological fracture sometimes happens after surgery in the coming weeks. Few authors advise this preventive/prophylactic mandibular plating (PMP) method when the scope of bone loss, cortical involvement, and a great possibility of mandibular fracture sometime after the initial surgery might occur [1,5,6,7,8,9,10]. It is different when plating is used when the fracture is not present, but this approach greatly decreases the risk of pathological fracture that could occur sometime after initial wisdom teeth removal with or without any cyst enucleation in its direct proximity. Figure 1 presents a case of a delayed right mandibular basis fracture a few weeks after initial wisdom teeth 48 removal (Primary etiological factor). Fracture was inadequately treated by local application of conservative intermaxillary fixation (MMF) for an additional 6 weeks. After this timeline, the patient was consulted at our ward. Regarding the clinical findings, at presentation the patient showed facial swelling, asymmetry, mandibular instability with malunion, and a fistula located distally to tooth 47. The imaging findings on CBCT (**A**,**B**) indicated loss of both cortices, bone asymmetry with swelling, and periosteal elevation (**C**,**D**). It is worth noting that without adequate bone stability, bone revision, debridement, and removal of odontogenic infection (teeth 47), as well as inadequate bone ORIF (open reduction and internal fixation), the patient developed a chronic OM. (**C**–**G**) present how bone inflammation spreads in time along the bone, causing the formation of granulation tissue, bone instability, progression of loss of bone over time, and presence of typical necrotic bone (**D**). Concerning the treatment, the patient was scheduled for a microbiological evaluation and later a procedure under general anesthesia that consisted of an intraoral approach combined with wound debridement, fistula removal, bone ostectomy, modified Obwegeser-Trauner approach, total bone osteoplasty, and the application of a dedicated angular Medartis Plate (Medartis, Basel, Switzerland) to secure and reduce the mandibular fracture (**H**). As for the outcome, after surgical debridement, stabilization, and targeted medical therapy with the use of heparin and anticoagulants with aimed antibiotics, the local inflammatory findings decreased. After a few months of additional revision, the second step of the surgery was conducted. A significant bone gap was grafted to support the bone healing and grant more bone within the healed fracture line as COM with bacterial contamination was not present in the second surgery. This example emphasizes how OM spreads because of pathological mandibular fracture, bone immobility, and granulation tissue formation over time. Significant bone loss after removal of tooth 48 led to mandibular bone instability and is prone to possible fracture. A co-present simultaneous infection from necrotic tooth 47 and inadequate wound debridement and care of the mandibular bone fracture in time was the key to OM formation. Without any significant stability after wisdom teeth removalwithout a possible PMP approach and inadequate usage of local intramaxillary fixation (MMF)a fracture occurred. In cases such as this, CBCT can better depict the extent of bone loss, delayed fracture risk, and postoperative bone healing than conventional two-dimensional imaging alone. In summary, the primary etiological factor in this case was the delayed mandibular fracture that developed after extraction of impacted tooth #48, whereas secondary contributing factors included insufficient initial stabilization, lack of early surgical revision and debridement, and persistence of an adjacent odontogenic infectious focus related to tooth #47. Together, these factors promoted progression toward chronic osteomyelitis. Definitive management required microbiological assessment, surgical debridement, fracture revision, and rigid fixation, followed by targeted medical therapy. This combined approach resulted in reduction in local inflammatory findings and clinical improvement, although staged reconstruction remained necessary because of the residual bone defect.

**Figure 2:**
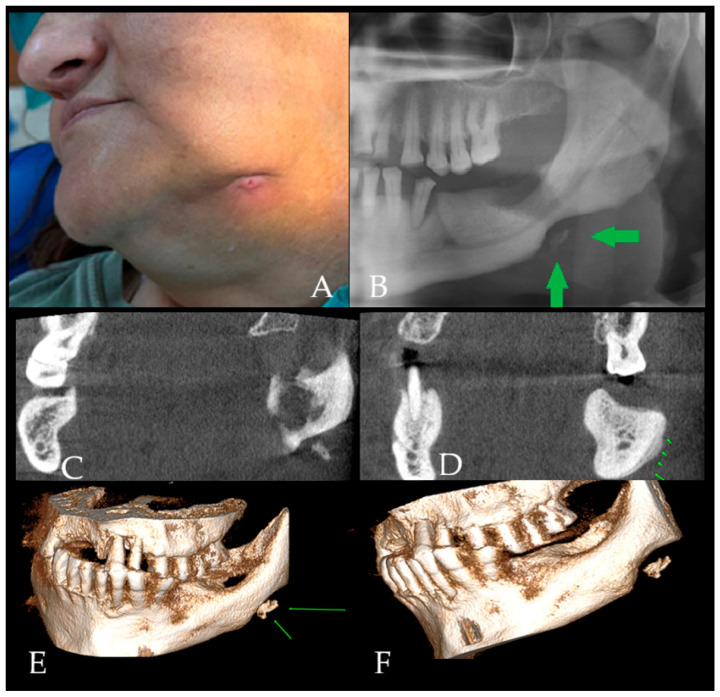
Chronic osteomyelitis with extraoral fistula and added diagnostic value of CBCT. OM is a potentially serious infection of bone tissue, most commonly caused by bacterial pathogens, with Staphylococcus aureus being the most frequently isolated organism in many series [1]. Infection may develop through hematogenous spread, direct inoculation (e.g., surgery/trauma), or contiguous spread from adjacent infected tissues (e.g., odontogenic infection), and it may lead to significant morbidity when it becomes chronic [1,2]. Despite advances in imaging and antimicrobial therapy, osteomyelitis remains clinically relevant because of its tendency toward persistence/recurrence, particularly when biofilm formation contributes to antibiotic tolerance and chronicity [2,3]. Clinically, the disease may present with swelling, pain, fistula formation, sequestrum, fever, trismus, and exposed bone; however, not all symptoms are present in every patient, particularly in chronic forms [4]. Facial asymmetry, swelling, severe pain, and skin irritation, as well as involvement of the oral mucosa, may indicate recurrent chronic osteomyelitis. Chronic systemic diseases, immunosuppression, and conditions that impair bone vascularity increase susceptibility to osteomyelitis [4,5,6]. In this case, a 52-year-old female patient presented with facial swelling and an extraoral fistula with discharge in the left mandibular region following the extraction of tooth #37; purulent drainage was evident on the left submandibular skin (**A**). The patient had a history of numerous systemic conditions, including coronary artery disease (CAD), pulmonary embolism, and diabetes, which are known risk factors for jaw osteomyelitis and its long-term inflammatory course [4,5,6]. The clinical impression at presentation was that of a chronic inflammatory process requiring radiological evaluation to determine bone involvement and exclude other aggressive lesions. Presence of such a fistula should be differentiated from lymphonodulitis, purulent lymph node, nodal cancer spread, skin lesions/inflammation, abscess, other metastatic carcinoma, as well as other bone-related conditions (ex., bisphosphonate-related, radiotherapy, and other). Osteomyelitis of the jaw occurs more frequently in the mandible than in the maxilla due to anatomical and physiological characteristics, including relatively limited mandibular blood supply and thicker, denser cortical bone [4,5]. Direct radiography remains a practical first step; however, panoramic imaging may be nonspecific, particularly in early or evolving disease, and can resemble a wide range of pathologies [7]. The initial dental panoramic radiograph (DPR) revealed an ill-defined osteolytic area in the posterior mandible body, extending from the crest to the base of the mandible (**B**). No contour abnormalities were observed in the mandibular canal. However, in the mandibular angle region, a notch-like destructive defect with contour irregularity was observed, together with radiopaque formations consistent with sequestration (Green arrow indicating bone sequestration). Trabecular disruption was observed along with a decrease in bone density in the left mandible molar region. Raising a broad differential diagnosis that included odontogenic infection-related bone changes, chronic inflammatory bone disease, and other jaw pathologies with aggressive radiological appearance [1]. At this stage, the dental panoramic radiograph established the presence of a destructive mandibular lesion but remained insufficient for full characterization. Although DPR suggested an ill-defined osteolytic process with possible sequestration, it could not reliably determine the true three-dimensional extent of bone involvement, the status of the buccal and lingual cortices, the presence and pattern of periosteal reaction, or the internal relationship between osteolytic, sclerotic, and sequestrated components. For this reason, CBCT was performed as the next diagnostic step. CBCT provided clear additional diagnostic value by demonstrating the exact extent of mixed osteolytic–sclerotic change within the left posterior mandible, sequestration at the mandibular base, cortical perforation of both buccal and lingual plates, and an onion-skin-type periosteal reaction. In contrast to DPR, the multiplanar and three-dimensional CBCT assessment allowed more precise evaluation of cortical destruction, trabecular disorganization, mandibular asymmetry, and the spatial distribution of the inflammatory process. These findings narrowed the differential diagnosis by favoring chronic osteomyelitis over other destructive jaw lesions and directly influenced clinical decision-making by supporting the need for surgical revision, debridement, and tissue sampling for histopathological confirmation rather than continued reliance on conventional imaging alone. The extent of bone swelling, asymmetry, atypical expansion, and other three-dimensional osseous changes should be carefully assessed on MDCT/CBCT (multidetector computed tomography/cone-beam computed tomography) to evaluate alterations in both cortical and trabecular bone. At the time of presentation, laboratory parameters, including leukocyte count, C-reactive protein, and erythrocyte sedimentation rate, were within normal limits. Chronic osteomyelitis (COM) is an inflammatory process developing in the medullary cavity or cortical surface of the bone and may be acute or chronic [5]. It is characterized by a complex inflammatory process involving necrosis of marrow and mineralized bone tissue, alongside pathological changes such as suppuration, resorption, sclerosis, and hyperplasia [8]. Involvement of the mandibular canal frequently results in pain, neural irritation, and exacerbation of local inflammation. Because DPR can underestimate the lesion’s extent and internal structure, MDCT/CBCT plays an important role in evaluating cortical integrity, trabecular architecture, and the presence of sequestra or sclerosis, as well as identifying potential sources of infection (teeth-related, periapical lesions, iatrogenic-based inflammation post previous surgery, dental treatment, or other) [7]. CBCT images in this case (**C**–**F**) demonstrated mixed osteolytic and sclerotic changes within the affected mandibular region. (**C**) Cone-beam computed tomography (CBCT) images revealed a sequestration area at the base of the left posterior mandible. (**D**) In addition, an “onion-skin” patterned periosteal reaction was observed in the buccal cortical bone of the affected area (green arrows show periosteal reaction). (**E**,**F**) 3D reconstruction showing an area of sequestration at the base of the left posterior mandible (green arrows indicate bone sequestration associated with chronic inflammation, consistent with features of chronic osteomyelitis). These radiological findings are consistent with bone remodeling due to a chronic infection process [5,8]. Cortical perforation was observed in buccal and lingual cortices, and the overall extent of bone involvement was more clearly delineated than on DPR. The 3D reconstruction further highlighted the distribution of cortical and trabecular changes and helped guide the surgical plan. Although imaging patterns in chronic osteomyelitis may suggest infection, overlap exists with other destructive jaw conditions; therefore, tissue diagnosis is important when imaging is atypical or the clinical course is prolonged [7]. In many cases, conventional radiography does not directly demonstrate cortical expansion, trabecular bone loss, mandibular asymmetry, or the full extent of osseous involvement within the affected region. Histopathological confirmation of this same case is presented in Figure 3.

**Figure 3:**
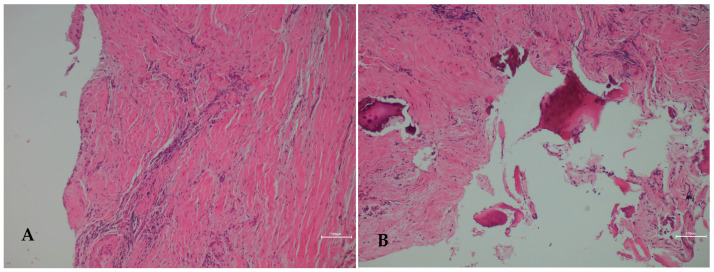
Histopathological confirmation of chronic osteomyelitis in a radiologically aggressive mandibular lesion. Bone biopsy and culture methods are considered the gold standard for definitive diagnosis of the etiological agent and for excluding other pathologies [7]. In chronic osteomyelitis cases, medical treatment alone is rarely sufficient, and combined surgical and medical management is typically required [8,9]. In the present case, surgical management consisted of local debridement, removal of sequestrated bone, curettage of the affected region, contour correction of the irregular alveolar crest, and excision of the extraoral fistula, followed by primary closure. Postoperative management included antibiotic, analgesic, and oral antiseptic therapy. Histopathological examination (Figure 3) revealed fibrous connective tissue areas demonstrating dense chronic inflammatory cell infiltration (**A**) (H&E, ×100 HPF). In addition, reactive bone fragments with irregular trabecular architecture were identified interspersed within the chronically inflamed fibrous stroma (**B**) (H&E, ×100 HPF). These findings were diagnostically important because they supported a persistent infectious-inflammatory process with reactive bone remodeling, which correlated well with the CBCT findings of mixed osteolytic-sclerotic change, sequestration, cortical disruption, and periosteal reaction described in Figure 2. In this way, the histopathological findings helped explain that the radiological appearance reflected chronic inflammatory bone destruction and repair rather than a primary odontogenic tumor or malignant bone lesion. Intraoperative cultures demonstrated polymicrobial growth, further supporting an infectious etiology, although without isolation of a single dominant pathogen. Therefore, the final diagnosis was established through integration of clinical presentation, CBCT features, microbiological findings, and histopathological confirmation. The presence of chronic inflammatory infiltration associated with reactive bone formation and remodeling was considered compatible with features favoring chronic osteomyelitis**.** These microscopic features supported a persistent infectious-inflammatory process rather than an odontogenic tumor or malignancy, resolving the primary diagnostic dilemma created by the aggressive radiological appearance [4,5,7,8]. Intraoperative cultures showed polymicrobial growth, precluding identification of a specific causative organism. Biopsy should always consist of evaluation of two or three different bone pieces to exclude any worrisome situations like osteosarcoma, other tumors, or the presence of bone cancer.

**Figure 4:**
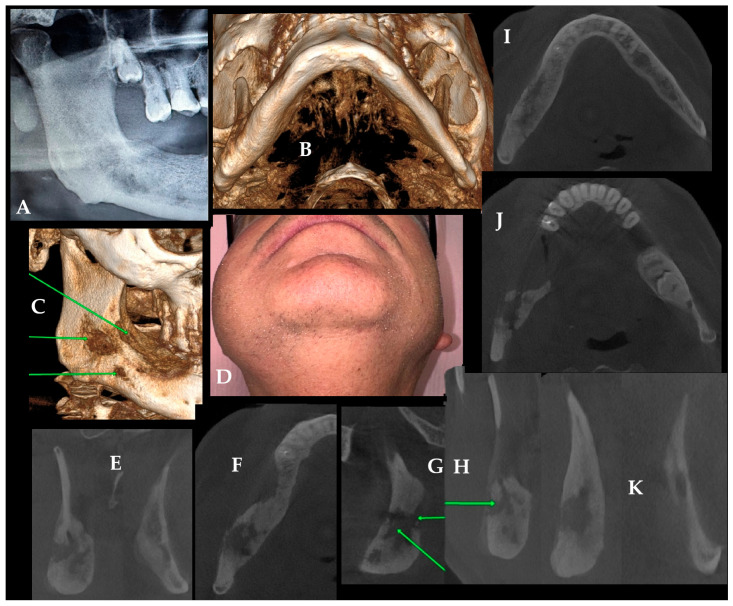
Long-standing chronic osteomyelitis with marked mandibular remodeling and clinicoradiological progression. Quite an important remark should address that chronic osteomyelitis (COM) may persist for many years and, in rare cases, can present clinical and radiological features that overlap with aggressive bone lesions, including osteosarcoma. Although such an association is extremely uncommon, this highlights the importance of close follow-up in patients with longstanding COM, particularly when progression or atypical findings are observed on clinical or radiological examination. In this case, wisdom teeth 48 with a cyst formation were removed more than ten years ago. Significant local bone inflammation was treated for years, without any sufficient bone revision, biopsy, or approach towards the bone asymmetry, swelling, facial pain, and presence of COM. It is quite important to understand that despite significant patient facial swelling (**D**), routine DPR (**A**) provided only limited radiological information and underestimated the extent of disease. In this case, DPR raised only a general suspicion of mandibular pathology, whereas CBCT clearly demonstrated cortical destruction, bone remodeling, periosteal reaction, and the true three-dimensional extent of the lesion. These additional CBCT findings were clinically important because they supported the decision for surgical revision, biopsy, and microbiological sampling rather than continued conservative management alone. In a routinely performed CBCT, loss of both cortices, mandibular angle asymmetry, and swelling with bone remodeling, suggested progression of COM over the years (**B**,**C**). Presented COM is quite similar to Garre-like osteomyelitis with a very long time of symptom occurrence (green arrows indicate irregular bone structure loss). Chronic pain, extensive swelling and some worrisome clinical symptoms forced the patient to seek help in our ward. Mandibular asymmetry with bone loss and remodeling (**E**–**J**) was present. A comparison of right-to-left mandibular angle (**K**) shows the scope of bone changes with remodeling, loss of its trabecular lining, and periosteal elevation. The patient was scheduled for a bone revision under general anesthesia. The procedure consisted of bone ostectomy, the Obwegeser-Trauner approach, bone saucerization, ostectomy, and debridement. The bone was shapeless, more osteoclerotic, with local softening, and presented a COM for a long period of time. Surgery consisted of bone re-shaping, modeling, and providing a lot of bone samples to confirm the COM and exclude any OSA formation, as well as to establish microbiological flora. Additional antibiotic bone-sponges were applied with anticoagulant therapy for ten days. Patient remains free of unwanted bone inflammation and healed with good outcomes. The residual bone following excision remains under observation, and the patient has been informed about the potential need for a second revision surgery and additional bone decortication if necessary. The presented COM case emphasizes that adequate early bone revision, irrigation, and surgical debridement immediately after wisdom tooth and cyst removal may lead to more favorable outcomes in the early time-frame. Local bone infections originating from wound irritation or untreated alveolitis may lead to chronic osteomyelitis (COM) if not managed in time, as presented in Figure 4. Antibiotic therapy alone should not be considered as the only approach to any bone complications without its surgical revision, debridement, ostectomy, and flap coverage.

**Figure 5:**
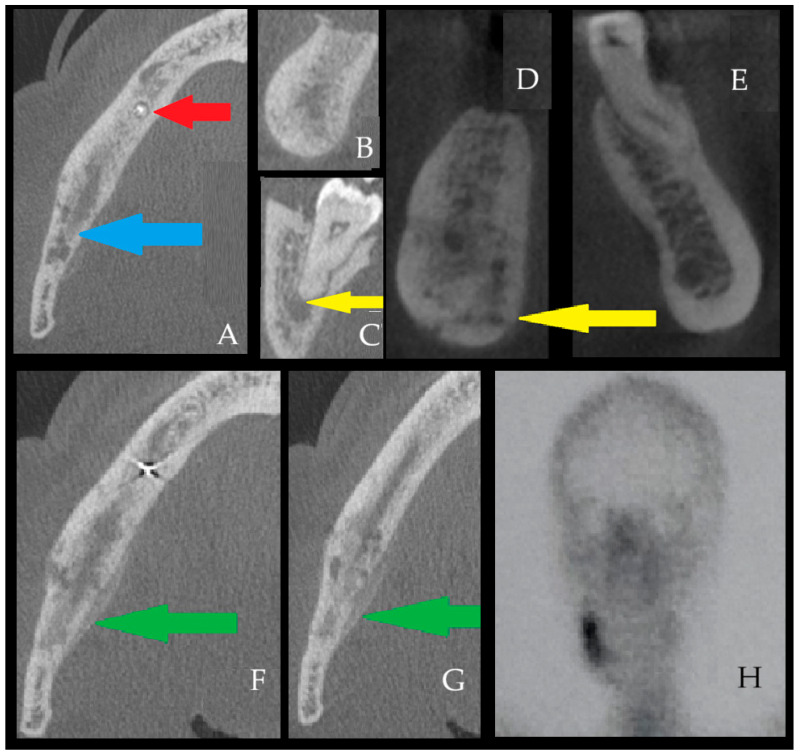
Garré-like secondary chronic osteomyelitis associated with persistent odontogenic infection. Other forms of bone inflammation, particularly long-standing or recurrent presentations, may suggest Garré osteomyelitis. First described by Carl Garré in 1893, this entity is also referred to as chronic recurrent osteomyelitis or sclerosing osteomyelitis of Garré [10,11,12,13]. This subtype of chronic osteomyelitis occurs predominantly in children and young adults and is most often linked to odontogenic infection and extensive dental foci. Most typically, it presents as localized overgrowth of bone in the external cortex and asymmetry, where the supracortical and subperiosteal parts of the bone are smooth, fairly calcified, and often seen and described as a duplication of the cortical layer of the mandible. Odontogenic infections may involve both the mandible and maxilla; however, sinus-related chronic infection, sometimes coexisting with dental sources, may also affect the maxilla [14,15,16,17]. In the presented Figure 5, a generally healthy 45-year-old woman was suffering from OM because of a complication that occurred after endodontic treatment of the tooth 46 periapical lesion (first lower right molar), which was not sufficiently treated and diagnosed in time. Due to the lack of adequate radiological and surgical diagnostics, insufficient antibiotic therapy (clindamycin) combined with teeth removal without surgical curettage and revision, this situation resulted in a progressive worsening of the patient’s condition. On axial CBCT images (**A**), the red arrow indicates the endodontically treated tooth #45 with a small periapical lesion, and postoperative changes near the mandibular canal following the extraction of tooth #46 are visible. The blue arrow shows irregular radiolucent bone deficiency common for the initial stages of osteomyelitis, loss of trabecular bone pattern, and presence of radiolucent irregular bone losses. (**B**,**C**) (CBCT coronal view of the right mandibular body) demonstrates asymmetry, inflammatory swelling, and granulation tissue formation. (**D**) demonstrates an irregular radiolucent area in the right mandibular body, consistent with early inflammatory bone changes and without periosteal reaction, that can be compared to the healthy left side (**D**,**E**). The sagittal CBCT view (**F**), the region posterior to the extracted tooth #46 shows a large periapical lesion with an active infectious focus beneath tooth #47, representing an additional odontogenic source in the absence of adequate endodontic therapy (yellow arrow, (**C**)-periapical lesion from tooth apex). During the period in which the patient sought adequate surgical consultation, her condition progressively worsened, and treatment with NSAIDs and antibiotics was ineffective. Over the subsequent two years, she underwent multiple dental interventions, pharmacological therapies, hyperbaric oxygen treatment, repeated courses of antibiotics, and prolonged NSAID use for persistent pain, without clinical improvement. (**F**,**G**)—CBCT views demonstrate bone swelling, cortical perforation, loss of cortico-cancellous borders, and an irregular osteolytic lesion with periosteal elevation from the lingual aspect (green arrow). Images present irregular borders and an osteolytic appearance of the right mandibular base compared with the healthy left side (**D**,**E**)-with visible periosteum elevation, yellow arrow, together with noticeable periosteal elevation and swelling (yellow arrow). (**F**,**G**) demonstrates condensing osteitis (Garré osteomyelitis), showing the characteristic radiologic appearance of Garré osteomyelitis (GOM). Typical spongy bone loss of trabecular build, shape, and structure between GOM from image (**D**,**E**) and healthy bone with preserved trabecular structure on the left mandibular basis. (**H**) Entire body SPECT-CT evaluation was scheduled to estimate the scope of bone infection and its inflammation as preparation for surgery. Any active chronic bone inflammation is considered the main source of the patient’s pain, mandibular swelling, toothache, and pain within the right inferior alveolar nerve, causing a dull pain within the right part of the lower lip. In this case, the final procedure was performed under general anesthesia. The patient underwent a modified Obwegeser–Trauner decortication with extraction of tooth #48, apicoectomy of tooth #47 (to preserve occlusion and prevent the Godon sign), decompression of the mandibular canal in the region of tooth #46, as well as bone curettage and ostectomy, followed by histopathologic and microbiological evaluation and placement of an antibiotic sponge. Histopathology concluded a chronic bone inflammation, while the bone scrub consisted of Prevotella media, Staphylococcus aureus, and Escherichia coli. Postoperative management included a 14-day antibiotic regimen and adjunctive supportive therapy for temporary inferior alveolar nerve dysfunction. After surgery and targeted antibiotic therapy with anticoagulants, the patient was free of pain, gingival and skin swelling, and local inflammation; however, residual bone changes were still visible and closely monitored for two years postoperatively. Swelling of the right mandibular body had decreased; however, complete bone healing comparable to the healthy left side was not yet evident. In contrast, bone decortication, ostectomy, and curettage resulted in a marked reduction in periosteal elevation and periosteal reaction when the two sides were compared. The presented case highlights that various imaging modalities, primarily DPR and computed tomography (CT) are used in the evaluation of jaw osteomyelitis (OM), osteoradionecrosis (ORN), Garre condensing osteomyelitis (GOM), and drug-induced jaw osteonecrosis (MRONJ/BRONJI) [3]. In addition, the diagnostic contribution of nuclear medicine techniques such as bone scintigraphy, SPECT/CT, and PET/CT has also been reported in the literature [4,5,8]. Elimination of odontogenic, sinus-related, odontogenic sinusitis, skin-related, or other potential sources of infection is mandatory [14,18,19,20,21]. A substantial body of literature exists regarding the imaging diagnosis of jaw osteomyelitis (OM), and studies indicate that MDCT is more sensitive than conventional two-dimensional radiography in detecting early abnormalities [6] and superior in demonstrating sequestra and periosteal reactions [7]. Furthermore, the volumetric data provided by CT allows for the evaluation of the lesion in different planes and three-dimensional analysis [3]. Based on these characteristics, Baba et al. [22] defined MDCT as the gold standard in the imaging diagnosis of jaw osteomyelitis (OM) due to its ability to accurately determine lesion size. Posadzy et al. [23] reported that the diagnostic performance of cone-beam computed tomography (CBCT) is comparable to that of multi-detector CT. On the other hand, Lee et al. [9] suggested that magnetic resonance imaging (MRI) is an effective method for early diagnosis of jawbone osteomyelitis and possibly confirms other non-bone-related lesions. When interpreted within the Zurich classification system proposed by Baltensperger and Eyrich [24], the presented cases are most consistent with secondary chronic osteomyelitis of odontogenic or iatrogenic-dental treatment-related origin, characterized by prolonged clinical course, cortical destruction, sequestration, fistula formation, and microbiologically confirmed infection. In the Garré-like case presented in Figure 5, the differential distinction between true Garré osteomyelitis (i.e., primary chronic osteomyelitis with proliferative periostitis) and secondary chronic osteomyelitis with periosteal reaction is particularly important. Although the radiological appearance showed a proliferative periosteal pattern reminiscent of Garré osteomyelitis, the overall profile of the case favored secondary chronic osteomyelitis rather than a true primary proliferative form. This interpretation was based on the adult age of the patient, the presence of a persistent odontogenic infectious source, progressive osteolysis, cortical perforation, prolonged symptomatic course, and microbiologically confirmed bacterial infection. Thus, in this case, the periosteal reaction was interpreted as a reactive manifestation of secondary odontogenic osteomyelitis rather than as an isolated defining feature of true Garré osteomyelitis. Taken together, these findings underscore that radiological phenotype alone may be misleading and that multimodal assessment integrating clinical history, imaging, microbiology, and histopathology is essential for accurate diagnosis and treatment planning in chronic osteomyelitis of the jaws. In summary, the radiographic findings showed good overall correlation with the clinical presentation, as the patient’s persistent pain, mandibular swelling, asymmetry, and inferior alveolar nerve-related symptoms corresponded to the CBCT evidence of cortical destruction, inflammatory bone expansion, periosteal reaction, and persistent odontogenic infectious foci. At the same time, the imaging findings also demonstrated that the disease was more extensive than might be assumed from the clinical examination alone, particularly with respect to trabecular disruption, cortical perforation, lingual periosteal involvement, and the presence of an additional active infectious source related to the adjacent tooth. Thus, in this case, CBCT not only supported the clinical suspicion of chronic mandibular osteomyelitis but also clarified the full anatomical extent and multifactorial odontogenic background of the disease, which was essential for definitive surgical planning and interpretation of the Garré-like radiological pattern.

**Figure 6:**
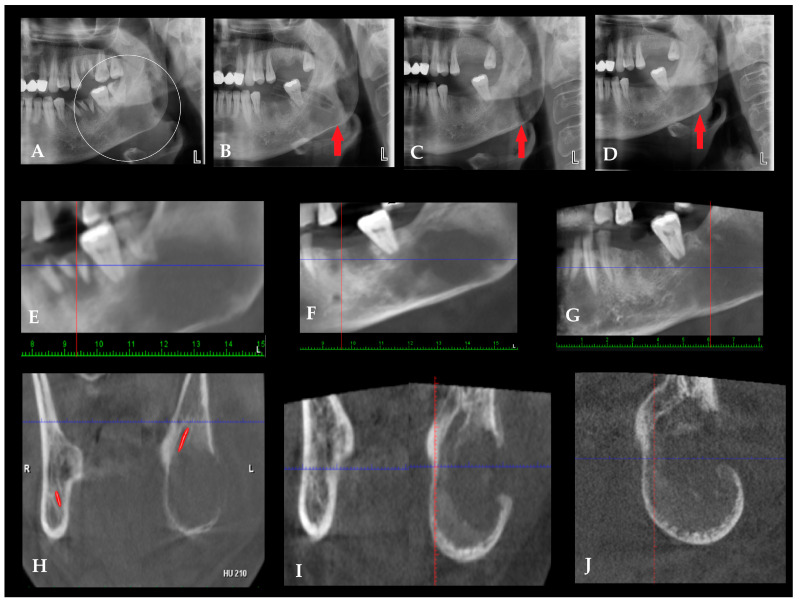
Secondary infection in fibro-osseous altered bone mimicking chronic osteomyelitis. Not all cases of chronic osteomyelitis (COM) of the jaws arise solely from odontogenic infections, iatrogenic procedures, or traumatic factors. In some situations, pre-existing bone conditions characterized by altered architecture and vascularity, such as fibrous dysplasia (FD), cemento-osseous dysplasia (COD), or other fibro-osseous lesions (FOLs), may predispose the affected bone to secondary infection [25,26,27]. The structurally abnormal bone present in these lesions may be more susceptible to bacterial colonization and local inflammatory processes when exposed to odontogenic infection or surgical trauma. Consequently, secondary infection of a fibro-osseous lesion may clinically and radiologically resemble chronic osteomyelitis. Conversely, chronic inflammatory bone changes may occasionally mimic the imaging characteristics of fibro-osseous lesions [28]. For this reason, careful clinical and radiological evaluation, particularly with CBCT, is essential to differentiate between primary COM, typical fibro-osseous lesions, and cases in which both conditions coexist. In all cases, potential odontogenic, jaw-related or sinus-related sources of infection should be thoroughly investigated. Fibro-osseous lesions of the jaws, particularly fibrous dysplasia (FD), may present with radiologic patterns that closely resemble chronic inflammatory bone disease, creating a diagnostic overlap that can be challenging when interpretation relies on a single imaging time point. FD is a benign developmental condition characterized by replacement of normal bone with fibro-osseous tissue, typically producing diffuse expansion, cortical thinning, and a heterogeneous internal “ground-glass” or mottled trabecular appearance [29]. These features may radiographically simulate chronic osteomyelitis when superimposed structural irregularities, trabecular rarefaction, or focal density variations are present. However, when infection is not superimposed, imaging features favoring FD/COD over chronic osteomyelitis include a more homogeneous ground-glass or mottled internal pattern, relative symmetry, lack of a clear odontogenic source, and the absence of sequestrum, fistula, or marked cortical destruction. In such situations, DPR alone may be insufficient because two-dimensional projection imaging can obscure subtle internal architecture and mask evolving changes within cancellous bone. Advanced imaging with CBCT enables more precise visualization of cortical contours, trabecular organization, and medullary structure, which is particularly valuable when lesions demonstrate atypical asymmetry or localized architectural disruption. Radiographically, odontogenic infection is more strongly suggested when the lesion is contiguous with a diseased tooth, periapical focus, periodontal defect, or post-extraction site, whereas non-odontogenic or fibro-osseous conditions are more often associated with diffuse expansion, generalized internal bone alteration, and a ground-glass or mottled appearance without a single clear dental source. In the present case, CBCT demonstrated features of both categories, namely a fibro-osseous-type internal architecture together with superimposed inflammatory change and cortical disruption, supporting the interpretation of secondary infection arising in previously altered bone. (**A**) Initial DPR obtained at presentation with an extraoral abscess demonstrating a heterogeneous radiolucent–radiopaque pattern within the left mandibular body and ramus region, characterized by trabecular disorganization and regional density variation. These findings suggest a fibro-osseous lesion while exhibiting radiologic features that may overlap with chronic inflammatory bone disease. (**B–D**) red arrow points radiolucent area in the angle, DPR obtained during treatment after placement of surgical drains, demonstrating persistence of the altered internal bone architecture. (**C**) Short-term follow-up DPR following surgical curettage showed partial radiologic stabilization of the lesion with persistent mottled internal structure. (**D**) Long-term follow-up DPR illustrating gradual bone remodeling while maintaining heterogeneous trabecular architecture. (**E**–**G**) where red and blue lines are orientation lines, Panoramic reconstructions derived from CBCT imaging provide improved visualization of lesion extent and internal bone structure compared with conventional two-dimensional panoramic imaging. (**H**–**J**) Coronal CBCT sections demonstrating buccolingual expansion of the mandibular ramus with cortical thinning and focal buccal cortical fenestration. Diffuse radiolucent changes and trabecular irregularity are present within the medullary bone. The inferior alveolar nerve canal is indicated by the red marker in (**H**). (**H**,**I**) Comparison with the contralateral ramus demonstrates asymmetry and localized architectural disruption, radiologic features that may mimic chronic osteomyelitis in the presence of fibro-osseous lesions. The presented images illustrate situations in which dental and iatrogenic factors affect the bone. When potential bacterial contamination of local alveolar tissues is not identified and treated in a timely manner, it may spread and involve the entire mandibular bone, leading to chronic osteomyelitis (COM). A commonly reported protocol includes intravenous antimicrobial therapy followed by oral therapy, together with sequestrectomy or decortication when indicated, along with anticoagulant medication [12]. Adjuvant measures such as hyperbaric oxygen therapy and local antibiotic delivery systems may provide additional benefit; however, current management primarily focuses on surgical debridement, revision, and curettage, sometimes combined with Obwegeser–Trauner decortication or bone saucerization [7,9,10,11,12,13,14,15]. Any mandibular osteomyelitis, particularly in medically complex patients, may present with striking clinical findings and nonspecific radiological patterns, requiring systematic correlation of history, imaging, and histopathology to avoid misdiagnosis and delay [4,5,7,8,9,10,11,12,13,14,15]. Typical and atypical bone changes seen in chronic osteomyelitis (COM), acute osteomyelitis (OM), and other osseous diseases are often nonspecific and may overlap with alternative pathologies. These features include periosteal elevation or thickening, sometimes exhibiting a sunburst or spiculated pattern reflecting reactive bone formation as well as cortical erosion or perforation, patchy osteolysis, mixed lytic–sclerotic areas, sequestrum formation, involucrum development, medullary sclerosis, and disruption of the inferior mandibular border. Additional findings may involve widening or displacement of the mandibular canal, loss of normal trabecular architecture, and regional bone expansion, all of which can mimic aggressive odontogenic tumors, fibro-osseous lesions, metastatic disease, or primary bone malignancies [16,17]. At the same time, CBCT has important diagnostic limitations, as the radiographic appearance of chronic osteomyelitis may overlap considerably with fibro-osseous lesions, malignant bone disease, and other destructive mandibular pathologies [30,31]. For this reason, imaging findings alone are insufficient in equivocal cases and should be correlated with clinical evolution, surgical findings, microbiology, and histopathology (Supplementary Table S1). Consequently, three-dimensional imaging with CBCT or MDCT, interpreted in conjunction with clinical presentation, microbiological data, and histopathological confirmation, remains essential for establishing the correct diagnosis and guiding timely surgical management.

## Data Availability

The data presented in this study are available on request from the corresponding authors due to patient privacy.

## Supplementary Materials

The following supporting information can be downloaded at: https://www.mdpi.com/article/10.3390/diagnostics16101427/s1, Table S1: Structured summary of the five illustrative cases, including diagnostic patterns, treatment approaches, diagnostic dilemmas, and key learning points.
