# Supplementary material for: How Clinical and Radiological Findings in Chronic Mandibular Osteomyelitis Do Not Always Correlate: Diagnostic Dilemmas in Dental-Related Bone Inflammations
Source: Diagnostics (Basel). 2026 May 7;16(10):1427. doi: 10.3390/diagnostics16101427 (PMC13205823; doi:10.3390/diagnostics16101427)
Supplement: Supplementary file 1 [file diagnostics-16-01427-s001.zip › diagnostics-4216684-supplementary.pdf]

This descriptive image-based report presents five illustrative patients across six figure-based sections, with Figures 2 and 3 referring to the same patient and emphasizing different stages of the diagnostic work-up. The cases were selected because they demonstrated clinicoradiological discordance, atypical radiological aggressiveness, or overlap with other mandibular pathologies. The purpose of this manuscript was not to compare imaging protocols or determine diagnostic accuracy, but to illustrate diagnostic dilemmas encountered in chronic mandibular osteomyelitis in routine clinical practice. Available imaging across the presented cases included dental panoramic radiography (DPR) and cone-beam computed tomography (CBCT), with multidetector computed tomography (MDCT) or SPECT/CT added only when clinically indicated. Image assessment focused descriptively on lesion location, cortical destruction or perforation, trabecular disorganization, sequestration, periosteal reaction, mandibular expansion/asymmetry, and relationship to possible odontogenic foci. Radiological suspicion of chronic osteomyelitis was based on the combination of these findings together with clinical history, whereas final case interpretation relied on clinicoradiological correlation and, where available, microbiological, intraoperative, and histopathological confirmation. A structured overview of the presented material is provided in Table S1.

Table S1. Structured summary of the five illustrative cases, including diagnostic patterns, treatment approaches, diagnostic dilemmas, and key learning points.

Patient	Case type	Final diagnosis	Shared / key features	Treatment	Diagnostic dilemma	Learning point
1	Delayed mandibular fracture after impacted third molar extraction	Secondary COM with delayed fracture and persistent odontogenic infection	Post-extraction bone loss, instability, fistula, cortical destruction, periosteal reaction, progressive bone loss, necrotic bone	Debridement, fistula excision, fracture revision, osteoplasty/ostectomy, plate fixation, targeted therapy; staged reconstruction planned	The main diagnostic challenge in this case was that delayed post-extraction mandibular fracture, persistent local infection, and progressive bone destruction together created a clinicoradiological picture of chronic osteomyelitis rather than an uncomplicated postoperative course.	In cases with major post-extraction bone loss and instability, early recognition of fracture risk, adequate debridement, and rigid stabilization are essential to prevent progression to chronic osteomyelitis.
2	COM with extraoral fistula	Secondary COM with sequestration and periosteal reaction	Facial swelling, extraoral fistula, comorbidities, mixed lytic-sclerotic lesion, sequestration, cortical perforation, onion-skin periosteal reaction, chronic course	CBCT assessment, surgical debridement/fistulectomy, histopathologic sampling, postoperative anti-infective and supportive therapy	The main diagnostic dilemma was that the initial dental panoramic radiograph suggested a destructive mandibular lesion but did not adequately define whether the process represented chronic osteomyelitis or	CBCT provided the decisive additional information by demonstrating sequestration, cortical perforation, and periosteal reaction, thereby refining the diagnosis and supporting the need for surgical revision and

Patient	Case type	Final diagnosis	Shared / key features	Treatment	Diagnostic dilemma	Learning point
					another aggressive jaw pathology.	histopathological confirmation.
3	Long-standing mandibular remodeling after previous third molar/cyst surgery	Long-standing COM with marked remodeling and asymmetry	Long course, asymmetry, pain, cortical destruction, periosteal reaction, remodeling	Revision, ostectomy/debridement, reshaping, sampling, adjunctive therapy, follow-up	The principal diagnostic challenge was that longstanding COM produced marked mandibular remodeling and progressive asymmetry, features that may overlap with other aggressive osseous lesions.	In long-standing cases, conventional panoramic radiography may underestimate disease severity, whereas CBCT better demonstrates cortical destruction, periosteal reaction, and true lesion extent, thereby guiding the decision for surgical revision and tissue diagnosis.
4	Garré-like secondary chronic osteomyelitis related to persistent odontogenic infection	Secondary chronic osteomyelitis with proliferative periosteal reaction (Garré-like pattern)	Pain, swelling, asymmetry, cortical perforation, periosteal elevation, persistent infection	Surgery, decortication, curettage/ostectomy, source control, histopathology/microbiology, targeted therapy	The radiological pattern raised the differential question of true Garré osteomyelitis versus secondary chronic osteomyelitis with periosteal reaction; however, the persistent odontogenic focus, adult presentation, cortical perforation, and microbiologically confirmed infection favored the latter.	CBCT is particularly valuable when clinical symptoms persist despite treatment, because it can reveal the full extent of cortical destruction, periosteal reaction, and additional odontogenic infectious sources that may not be evident clinically alone.
5	Secondary infection in fibro-osseous altered bone	Secondary infection superimposed on fibro-osseous altered mandibular bone, mimicking chronic osteomyelitis	Mixed internal pattern, expansion, cortical thinning, trabecular disorganization, asymmetry	Curettage/debridement, drainage, follow-up, clinicopathological correlation, supportive therapy	The major challenge in this case was distinguishing secondary infection in fibro-osseous altered bone from primary COM since both may produce overlapping clinical and radiological findings.	When bone architecture is already altered by fibro-osseous disease, CBCT helps identify the distribution and character of the lesion, but final interpretation still requires integration of imaging with clinical findings, disease evolution, and histopathological assessment.

Abbreviations: CBCT, cone-beam computed tomography. COM, Chronic Osteomyelits
